# Role of the Type VI Secretion System in the Pathogenicity of *Pseudomonas syringae* pv. *actinidiae*, the Causative Agent of Kiwifruit Bacterial Canker

**DOI:** 10.3389/fmicb.2021.627785

**Published:** 2021-02-19

**Authors:** Nana Wang, Ning Han, Runze Tian, Jiliang Chen, Xiaoning Gao, Zhiran Wu, Yuqi Liu, Lili Huang

**Affiliations:** ^1^State Key Laboratory of Crop Stress Biology for Arid Areas, Northwest A&F University, Yangling, China; ^2^College of Life Science, Northwest A&F University, Yangling, China; ^3^College of Plant Protection, Northwest A&F University, Yangling, China; ^4^Institute of Bioengineering, Guangdong Academy of Sciences, Guangzhou, China

**Keywords:** pathogenicity, type VI secretion system, Kiwifruit bacterial canker, type III secretion system, competition, biofilm formation, environmental adaptability

## Abstract

The type VI secretion system (T6SS), a macromolecular machine, plays an important role in the pathogenicity of many Gram-negative bacteria. However, the role of T6SS in the pathogenicity of *Pseudomonas syringae* pv. *actinidiae* (*Psa*), the pathogen of kiwifruit bacterial canker, is yet to be studied. Here, we found a T6SS gene cluster consisting of 13 core genes (A-J) in the genome of *Psa* M228 based on a genome-wide analysis. To determine whether the T6SS gene cluster affects the pathogenicity of *Psa* M228, T6SS and its 13 core gene deletion mutants were constructed and their pathogenicity was determined. The deletion mutants showed different degrees of reduction in pathogenicity compared with the wild-type strain M228; in *tssM* and *tssJ* mutants, pathogenicity was significantly reduced by 78.7 and 71.3%, respectively. The pathogenicity results were also confirmed by electron microscopy. To further confirm that the reduction in pathogenicity is related to the function of T6SS, we selected the T6SS gene cluster, comprising *tssM* and *tssJ*, for further analyses. Western blot results revealed that *tssM* and *tssJ* were necessary for hemolytic co-regulatory protein secretion, indicating that they encode a functional T6SS. Further, we explored the mechanism by which T6SS affects the pathogenicity of *Psa* M228. The ability of bacterial competition, biofilm formation, hydrogen peroxide tolerance, and proteolytic activity were all weakened in the deletion mutants M228ΔT6SS, M228ΔtssM, and M228ΔtssJ. All these properties of the two gene complementation mutants were restored to the same levels as those of the wild-type strain, M228. Quantitative real-time results showed that during the interaction between the deletion mutant M228ΔT6SS and the host, expression levels of T3SS transcriptional regulatory gene *hrpR*, structural genes *hrpZ*, *hrcC*, *hopP1*, and effector genes *hopH1* and *hopM1* were down-regulated at different levels. Taken together, our data provide evidence for the first time that the T6SS plays an important role in the pathogenicity of *Psa*, probably via effects on bacterial competition, biofilm formation, and environmental adaptability. Moreover, a complicated relationship exists between T6SS and T3SS.

## Introduction

The bacterial canker of kiwifruit is caused by the virulent form of *Pseudomonas syringae* pv. *actinidiae* (*Psa*) and is the most prevalent disease in the kiwifruit industry (Pinheiro et al., 2020). *Psa* can enter the plant through stomata, water holes, lenticels, wounds (caused by birds, insects, and/or human contact) and can colonize the plant (including branches, leaves, buds, leaf marks, and pruned diseased branches) for a long time without inducing external symptoms, until a suitable environmental condition is developed (Donati et al., 2020). Systemic infection occurs through various pathways (Stefani and Giovanardi, 2011; Tontou et al., 2014). *Psa* can infect young twigs systemically within a few minutes (Michelotti et al., 2018). Therefore, pathogenic bacteria can infect kiwifruits repeatedly, breed in cortex, expand up and down, and even move to the xylem and central column of the plant, causing severe pathogenicity under appropriate conditions (Gao et al., 2016). Due to the latent and short-term outbreak characteristics of *Psa*, bacterial canker has been identified as a destructive disease of kiwifruit, which results in production losses worldwide. Recently, this disease has received more attention in the main kiwifruit planting areas owing to the huge economic losses (Vanneste, 2017; Hang et al., 2018; Kim et al., 2019; Williams et al., 2020). Therefore, identifying the pathogenic mechanism will play a vital role in effective prevention and control of the disease.

Bacterial phytopathogens usually infect their host plants by various extracellular proteins or secretion of effectors through secretory systems (Khan et al., 2018; Deb et al., 2019). These secretion systems are a class of complex nanomolecular machines that can transport the virulence proteins to the external environment or host cells directly or indirectly (Costa et al., 2015; Wen et al., 2018). To date, at least six different types of secretion systems (I–VI) have been identified in Gram-negative pathogenic bacteria (Mcquade and Stock, 2018). Among them, a newly discovered secretion system, the type VI secretion system (T6SS), has been shown to play an important role in pathogenicity (Agnetti et al., 2019; Zhu et al., 2020) and has also been implicated in bacterial interactions or environmental adaptations such as colonization, biofilm formation, resistance, and survival (Mathias et al., 2017; Wu et al., 2018; Ben-aakov and Salomon, 2019; Asolkar and Ramesh, 2020). T6SS comprises 13 core genes (*tssA* to *tssM*) usually encoded within the same gene cluster (Leiman et al., 2009; Catarina et al., 2011; Kapitein et al., 2013; Kudryashev et al., 2015; Cianfanelli et al., 2016). Among them, hemolytic co-regulatory protein (Hcp) of bacteriophage T4 (Pell et al., 2009; Nguyen et al., 2018) is one of the major substrates secreted by T6SS. Since the secretion of Hcp is T6SS-dependent, it is often a reliable indicator of whether T6SS functions appropriately (Pukatzki et al., 2009).

Multiple studies have found that T6SS was closely related to the pathogenicity in many pathogenic bacteria strains. Mattinen et al. (2010) found that there was four H in the extract from pathogenic bacterium *Pectobacterium atrosepticum*, when it was cultured in minimal medium supplemented with host extract. Overexpression mutants of the *hcp* genes were constructed. The pathogenicity of the mutant strains was enhanced compared with that of the wild-type strain. Hcp may act as a new virulence factor in *P. atrosepticum* (Mattinen et al., 2010). Since the secretion of Hcp is dependent on the T6SS secretion system, the results indicates that T6SS plays an important role in *P. atrosepticum*. The crown gall disease caused by *Agrobacterium tumefaciens* is a worldwide tumor forming disease of the plants and is a major problem for plant nursery industries. It can cause great economic loss in fruit plants (Habbadi et al., 2017). Wu et al. (2018) found that the pathogenicity of *A. tumefaciens* is closely related to T6SS. *Pantoea ananatis* LMG 2665 is the most prevalent pathogenic bacterium found in pineapple fruit and onions. Shyntum et al. (2015) found that two sets of T6SS in its genome, namely T6SS-1 and T6SS-3. T6SS-1 is closely related to the pathogenicity and competitiveness of the strain *P. ananatis* LMG 2665, while T6SS-3 has no effect on its pathogenicity. This indicates that T6SS-1 is an important virulence factor in *P. ananatis*. In addition, the core genes *tssM* (Zhang et al., 2012) and *tssB* (Zhang et al., 2014) of T6SS are essential for the pathogenicity of *Ralstonia solanacearum*. This indicates that T6SS plays an important role in an increasing number of plant bacterial diseases, where the core genes of T6SS have different functions. The bacterial canker of kiwifruit, caused by *Psa*, is the most prevalent disease in the kiwifruit industry; however, the function of T6SS in the pathogenicity of *Psa* is still unclear. In this study, we found a T6SS gene cluster consisting of 13 core genes (A-J) in the genome of *Psa* M228 based on a genome-wide analysis. We studied the role of T6SS in the pathogenicity of *Psa* by mutant construction through homologous recombination technology and conducted a preliminary exploration of its mechanism. The results showed that T6SS is an important pathogenic determinant in *Psa* M228 and plays a role in bacterial competition, biofilm formation, hydrogen peroxide tolerance, proteolytic ability, and T3SS function. Our results provide insights into the functions of T6SS in the pathogenic mechanism of *Psa*.

## Materials and Methods

### Bacterial Strains, Plasmids, and Reagents

*Pseudomonas syringae* pv. *actinidiae* strain M228 (*Psa* M228) was isolated from kiwi leaves in the Meixian, Shaanxi Province of China in 2010 (Gao et al., 2016). Deletion and complementation mutants were constructed in this study. The strain *Psa* M228 and mutants were grown on Luria-Bertani (LB) agar plates or cultured in LB liquid medium at 25 ± 1°C. *Escherichia coli* DH5α was grown in LB medium at 37°C. For gene cloning, *E. coli* S17-1λpir was cultured under the same conditions for bacterial gene transfer. When necessary, ampicillin (Amp, 10 μg/mL), kanamycin (Km, 10 μg/mL), tetracycline (Tcr, 10 μg/mL), erythromycin (Em, 10 μg/mL), and nalidixic acid (NAL, 10 μg/mL) were used. All strains used in this study were provided by the Plant Pathology Laboratory at Northwest A&F University, Yangling, and PRC. All reagents and solvents were of analytical grade. Antibiotics were purchased from Sigma (United States). Plasmid pK18mobsacB was used for gene deletion, and purchased from Biovector Science Lab, Inc. (Beijing, China); pDSK-GFPuV was used for fluorescently labeled strains construction, and was provide by Ph.D. Mysore (The Samuel Roberts Noble Foundation, United States); pMarA was used for *Bacillus* transformation, and provided by Professor Qi Wang (China Agricultural University, Beijing, China); pDSK was used for gene complementation, pBR322 was used for *E. coli* DH5α transformation, and these two plasmids were provided by Professor Xi-Hui Shen (Northwest A&F University, Yangling, China).

### T6SS Bioinformatics Analysis of *Psa* M228

The whole genome of *Psa* M228 has been sequenced by our laboratory, and the genome data have been registered on NCBI (ANJI00000000.2). The gene *tssC* sequence of *Psa* M228 was obtained through bioinformatics analysis, and an evolutionary tree was constructed from the *tssC* sequence of 68 species in 38 genera that contain T6SS. There are three representative types of T6SS and from the evolutionary tree; the species containing them were selected. Subsequently, a phylogenetic tree was constructed to determine the type of T6SS *Psa* M228 belonged to. The evolutionary history was inferred using the Maximum Likelihood method based on the Le_Gascuel_2008 model (Le and Gascuel, 1993). The bootstrap consensus tree inferred from 1000 replicates was used to represent the evolutionary history of the taxa analyzed (Felsenstein, 1985). Branches corresponding to partitions reproduced in less than 60% bootstrap replicates were collapsed. The percentage of replicate trees in which the associated taxa clustered together in the bootstrap test (1000 replicates) is shown next to the branches (Felsenstein, 1985). The initial tree(s) for the heuristic search were obtained automatically by applying Neighbor-Join and BioNJ algorithms to a matrix of pairwise distances estimated using a JTT model, and the topology with a superior log likelihood value was selected. A discrete Gamma distribution was used to model evolutionary rate differences among sites [five categories (+G, parameter = 2.4316)]. The analysis involved 34 amino acid sequences. All positions containing gaps and missing data were eliminated. A total of 443 positions in the final dataset was obtained. Evolutionary analyses were conducted in MEGA6 (Tamura et al., 2013).

According to the phylogenetic tree, the T6SS structural genes of *Psa* M228 were annotated. The database of Clusters of Orthologous Groups of proteins (COGs) was obtained from the National Center of Biotechnology Information^1^. A schematic diagram of the T6SS structure was constructed using the *Pseudomonas aeruginosa* PAO1 gene structure map as a reference (Sana et al., 2016).

### Generation of *Psa* M228 Mutant Strains

Mutant strains with deletions of T6SS and its 13 core genes were constructed by the homologous recombination method (Kvitko and Collmer, 2011). In brief, PCR primer synthesis and DNA sequencing were performed by TsingKe Biotech Co., Ltd. (Beijing, China). *Psa* genomic and plasmid DNAs were isolated using an E.Z.N.A. TM Bacterial DNA Kit (OMEGA, United States) and Plasmid Mini Kit I (OMEGA, United States), respectively. Restriction enzymes were purchased from TaKaRa (Dalian, China). Deletion mutants were constructed using the suicide vector pK18mobSacB (Schafer et al., 1994). The gene fragments including their left and right arms were amplified using the corresponding primers (Supplementary Table S1). The DNA fragments were digested with *Eco*RI*/Hin*dIII and ligated to pK18mobsacB. The vector pK18mobsacB carrying each target gene fragment was inserted by the Km resistance gene and deletion vectors were constructed. Then, the vectors were introduced into the M228 strain by conjugal mating experiments. The first recombinant mutants were screened on LB (Km10-NAL10-Amp 10 μg/mL) and PCR detected using primers SacB-F/R (Supplementary Table S2). The mutant candidates were then screened to remove the suicide plasmid, using LB agar plates containing 20% sucrose without sodium chloride. The results of target gene deletion were confirmed by PCR amplification and DNA sequencing.

### Complementation of *Psa* M228ΔtssM and M228ΔtssJ in Deletion Mutants

The complement mutants were constructed as follows: primers TssM-C-F/R and TssJ-C-F/R (Supplementary Table 1) were used to amplify the full-length *tssM* (3849 bp) and *tssJ* (474 bp) genes from the *Psa* M228 genome, as well as the upstream putative promoters. The PCR products were cloned into the *Bam*HI and *Nde*I sites of plasmid PDSK, and the plasmids were transformed into *E. coli* DH5α. The complementation plasmids PDSK-tssM and PDSK-tssJ were extracted from *E. coli* DH5α according to the manufacturer’s instructions in the Plasmid Mini Kit I (OMEGA, United States), and then electroporated into mutants M228ΔtssM and M228ΔtssJ, respectively. The complement mutants were screened by kanamycin resistance on LB agar.

### Spread Assays

The spreading ability of the strain *Psa* M228 and its mutant M228ΔT6SS was determined as previously described (Gao et al., 2016). In brief, the strains *Psa* M228 and its mutant M228ΔT6SS were successfully transformed with pDSK-GFPuV by electroporation (Huang et al., 2013). Kiwi branches (cv. “Hongyang”) were surface-disinfected by rinsing with running water, soaked in 0.6% sodium hypochlorite solution for 5 min, and rinsed with sterile water again. The base of the petiole was wrapped with absorbent cotton to avoid evaporation. Punctures were made at the main leaf vein 1–2 cm away from the petiole, and GFPuv labeled bacterial culture of 10 μL (diluted to OD_600_ = 0.1, concentration = 1 × 10^8^ CFU/mL) were dripped onto the wound. The inoculated materials were placed in an artificial climate incubator (photoperiod L/D: 16/8 h; day and night temperature: 16/4°C, relative humidity 95%). The results were observed using a stereo fluorescence microscope (Leica MZ10F, Leica Microsystems, Germany) after 2 days.

### Pathogenicity Assays

Pathogenicity of the strain *Psa* M228 and its mutants was determined in kiwi (cv. “Hongyang”), as described previously (Zhao et al., 2019). Healthy branches of kiwi (cv. “Hongyang”) were cut into short branches of about 15 cm each and rinsed with sterile water; the ends were sealed with paraffin to avoid evaporation. In each test, a wound deep to the phloem within a width of 2 mm was cut with a blade in the middle of the branch, and 10 μL of the substance (OD_600_ = 0.1, the concentration = 1 × 10^8^ CFU/mL) was inoculated, while using sterile water as a negative control. Each strain was inoculated with five branches, and each experiment was repeated three times. The branches were maintained in an artificial climate incubator at a temperature of 25°C under natural day and night cycles. The lesion lengths of the branches were measured after 30 days. The lesion length is the average of three replicates. Statistical significance was determined by Student’s *t*-test.

### Electron Microscopic Observation

Healthy leaves from kiwifruit (cv. “Hongyang”) were inoculated separately with bacterial suspensions of *Psa* M228 and its mutant strains M228ΔtssM and M228ΔtssJ, using the same method as described in section “Pathogenicity Assays.” Scanning electron microscopy (SEM) samples were prepared by taking the leaf blade (5–7 mm) from the edge of sick and healthy junctions after 48 and 96 h. Carefully processed as described by Kang (1995), the colonization of bacterial pathogens in leaf tissue was observed using a JSH 6360 SEM (JEOL Ltd., Tokyo, Japan) at 15 kV. Transmission electron microscope (TEM) samples from 96 h culture were prepared in the same way as described by Kang (1995), and alterations in the ultrastructure of the leaf tissue infested by pathogenic bacteria were observed using an HT7700 TEM (HITACHI Company, Tokyo, Japan).

### Western Blot Assays

To explore whether the T6SS gene cluster encodes a functioning T6SS, Hcp secretion was assayed by western blot. The wild-type strains *Psa* M228 and mutants M228ΔT6SS, M228ΔtssM, and M228ΔtssJ were cultured as described in section “Electron Microscopic Observation.” The supernatant and cells were obtained by centrifugation at 13,000 × *g* for 10 min at 4°C. The protein content was determined using a Micro BCA^TM^ Protein Assay Kit (Thermo Fisher Scientific, United States). Extracellular and intracellular proteins were subjected to 12% SDS PAGE and then transferred to PVDF membranes. The immunoblot analysis was performed using anti-VSV-G-tag monoclonal antibody (VSVG) (CB100151, Cali-Bio, United States) and anti-RNA polymerase (RNAP) antibody (W0023, NeoClone, Beijing, China) at a dilution of 1:1000 as the primary and secondary antibodies were goat anti-mouse horseradish peroxidase (HRP) (DY60203) (DIYI BIO TECHNOLOGY, Shanghai, China) and goat anti-rabbit HRP (DY60202) (DIYI BIO TECHNOLOGY, Shanghai, China), respectively, at a dilution of 1:10,000. The differences between the bands of intracellular and extracellular proteins were compared between M228 and the mutants, using RNAP as a control.

### Bacterial Competition Assays

*Escherichia coli* DH5α was transformed with plasmid pBR322 to confer tetracycline resistance and *Bacillus* was transformed with plasmid pMarA to confer erythromycin resistance used for the competition studies. The competition assays were carried out as per the protocol described by MacIntyre et al. (2010). Competitor strains were grown overnight in LB broth supplemented with tetracycline (10 μg/mL) or erythromycin (10 μg/mL). The cells were centrifuged and washed twice with fresh sterile LB broth (10,000 × *g*, 1 min). The washed cells were resuspended in LB broth (OD_600_ = 0.1, concentration = 1 × 10^8^ CFU/mL) and combined with the *Psa* M228 wild-type or mutant strain M228ΔT6SS at a ratio of 1:1. The mixture was cultured at 25°C for 24 h, and then spotted on LB agar plates and incubated at 30°C for 12 h for the competition strain bacterial analysis.

### Biofilm Assays

Biofilm assays were performed using the method described by Stepanovic et al. (2000). The results were determined by measuring the absorbance at 570 nm (OD_570_) using a Thermo Multiskan EX Micro plate Photometer (Thermo Fisher Scientific Inc., United States). The experiments were repeated three times with six replicates per treatment. The absorbance at 570 nm was the average of six replicates. Statistical significance was determined by Student’s *t*-test.

### Environmental Adaptability Assays

The hydrogen peroxide (H_2_O_2_) tolerance and proteolytic ability were evaluated in accordance with previously described procedures (Molina et al., 2005). The bacterial solutions (diluted to OD_600_ = 0.1, concentration = 1 × 10^8^ CFU/mL) were inoculated into LB broth medium containing H_2_O_2_ solution (10 mL, 0.06 mol/L) at a ratio of 1:100. Bacterial growth was measured at 600 nm absorbance every 12 h.

A total of 2 μL of the bacterial solution (diluted to OD_600_ = 0.1, concentration = 1 × 10^8^ CFU/mL) was inoculated in the acid hydrolyzed case in a medium plate (1%w/v). Colony diameter was measured after culturing at 25°C for 14 days. All assays were repeated three times, and each treatment was performed in triplicate. Statistical significance was determined by Student’s *t*-test.

### Quantitative Real-Time Assays

Healthy branches from kiwifruit (cv. “Hongyang”) were prepared as described in section “Pathogenicity Assays.” Suspension of *Psa* M228 and mutant M228ΔT6SS (diluted to OD_600_ = 0.5, concentration = 5 × 10^8^ CFU/mL, 10 μL) were inoculated into the wound made in advance, and cultivated in an artificial climate incubator (the illumination time was 16 h and the dark time was 8 h). The bark of the inoculated wound (deep to phloem) was taken at 2 and 16 h after inoculation. Operations for bacterial RNA extraction, cDNA synthesis, and qPCR were performed as previously described (Broms et al., 2009). The reference genes were *gyrB* (DNA gyrase subunit B), *dusA* (tRNA dihydrouridine synthase), and *ftrA* (transcriptional regulator) (Hirose et al., 2020). Data were analyzed following the protocol of the comparative critical threshold (CT) method (Zhang et al., 2014). The expression levels of the target genes (*hrpR*, *hrpZ, hrcC*, *hopP1, hopH1*, and *hopM1*) at different inoculation times were calculated relative to endogenous control genes using the relative quantification method (Lopez and Pardo, 2005). Quantitative data were presented as fold change mutant strain M228ΔT6SS compared to wild-type (WT) M228 levels at 2 and 16 h. Error bars represent the standard deviation of the ΔΔC value. Statistical significance was determined by Student’s *t*-test. The quantitative real-time PCR (qRT-PCR) was performed in triplicate for technical replicates.

## Results

### T6SS Gene Structure Diagram of *Psa* M228

**FIGURE 1:**
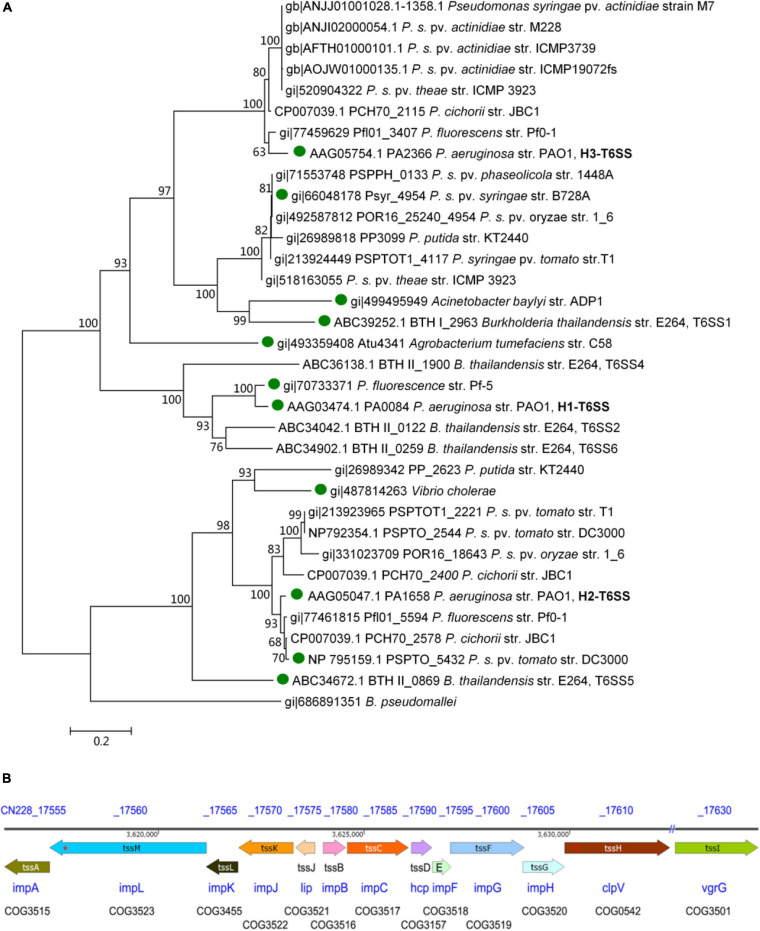
Structure of T6SS gene cluster analysis of the pathogenic strain *Psa* M228. **(A)** Molecular phylogenetic analysis by Maximum Likelihood method. The evolutionary history was inferred using the Maximum Likelihood method based on the Le_Gascuel_2008 model (Le and Gascuel, 1993). The bootstrap consensus tree inferred from 1000 replicates was used to represent the evolutionary history of the taxa analyzed (Felsenstein, 1985). Branches corresponding to partitions reproduced in less than 60% bootstrap replicates were collapsed. The percentage of replicate trees in which the associated taxa clustered together in the bootstrap test (1000 replicates) is shown next to the branches (Felsenstein, 1985). The initial tree(s) for the heuristic search were obtained automatically by applying Neighbor-Join and BioNJ algorithms to a matrix of pairwise distances estimated using a JTT model, and the topology with a superior log likelihood value was selected. A discrete Gamma distribution was used to model evolutionary rate differences among sites [five categories (+G, parameter = 2.4316)]. The analysis involved 34 amino acid sequences. All positions containing gaps and missing data were eliminated. A total of 443 positions in the final dataset was obtained. Evolutionary analyses were conducted in MEGA6 (Tamura et al., 2013). According to the results of the phylogenetic tree, one complete T6SS was found in *Psa* M228 and belonged to the same branch as that of the *P. aeruginosa* PAO1 H3-T6SS (HIS-III) gene cluster. “
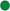
” indicates the functional T6SS that has been reported. **(B)**. Schematic diagram of theT6SS gene cluster of the pathogenic strain *Psa* M228. The name of the core genes of T6SS in Psa M228 are indicated by arrows. The direction of the arrows represents the direction of transcription of the genes in the genome. “//” indicates the presence of other genes not belonging to T6SS. “*” indicates the presence of frameshift mutation. The gene products are shown below the arrows. The database of Clusters of Orthologous Groups of proteins (COGs) was obtained from the National Center of Biotechnology Information (see text footnote 1).

According to the results of the phylogenetic tree constructed from the *tssC* sequence of 68 species in 38 genera (Supplementary Figure 1), and its simplified phylogenetic tree (Figure 1A), one complete T6SS was found in *Psa* M228 and belonged to the same branch as the *P. aeruginosa* PAO1 H3-T6SS (HIS-III) gene cluster. Then, the T6SS gene structure diagram in M228 was obtained.

As shown in Figure 1B, there are 13 conserved structural genes such as *tssA*-*tssM* necessary for T6SS in M228. The COGs of the cluster are impA, impB, impC, hcp, impF, impG, impH, clpV, vgrG, lip, impJ, impK, and impL, all of which are located in the M228 genome sequence contig054. Among them, *tssB* and *tssC* are the needle-sheath structural genes, *tssD* is the needle-tube structural gene, and *tssM*, *tssJ*, and *tssL* are transmembrane structural genes. However, there is a frameshift mutation in the transmembrane structural protein gene *tssM* (1272 codons) and the ATP hydrolase gene *tssH* (854 codons) (Figure 1B).

### Construction of T6SS Gene Cluster and 13 Core Genes Deletion Mutants in *Psa* M228

The previous sequence analysis showed that there is a T6SS gene cluster in the *Psa* M228 genome, which consists of 13 core genes (*tssA*-*tssM*). The function of the gene cluster and core genes in *Psa* has not been studied yet. Thus, in this study, we began by deleting the T6SS gene cluster and its individual 13 core genes of *Psa* M228 using λ Red-recombineering technique (Kvitko and Collmer, 2011), yielding strains M228ΔT6SS and 13 mutants from M228ΔtssA to M228ΔtssM.

### T6SS of *Psa* M228 Is Required for Spreading

**FIGURE 2:**
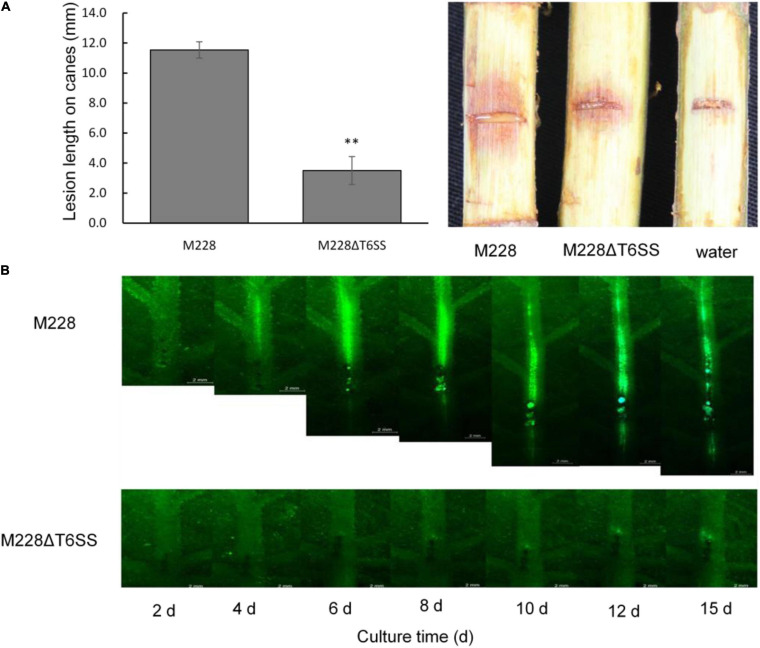
Pathogenicity and spreading ability analysis of the pathogenic strain M228 and its mutants M228ΔT6SS. **(A)** Pathogenicity analysis. The pathogenicity of the mutant M228ΔT6SS mutant was reduced by 69.65% compared with that of the wild-type strain. The lesion length is the average of five replicates. Statistical significance was determined by Student’s *t*-test. “**” Means the difference is significant at the 0.01 level. **(B)** Spreading ability analysis+. The spreading ability of mutant M228ΔT6SS in leaf veins was significantly different from that of wild-type M228. The spreading range of M228 in leaf veins increased with an increase in incubation time, and fluorescence began to weaken after 12 days. However, the spreading ability of M228ΔT6SS in leaf veins was significantly weakened, and it failed to expand rapidly in leaf veins, and only showed weak fluorescence near the inoculation point.

The spreading ability of mutant M228ΔT6SS in leaf veins was significantly different from that of wild-type M228. The spreading range of M228 in leaf veins increased with an increase in incubation time, and fluorescence began to weaken after 12 days. However, the spreading ability of M228ΔT6SS in leaf veins was significantly weakened; it failed to expand rapidly in the leaf veins and indicated only weak fluorescence near the inoculation point (Figure 2B).

### T6SS of *Psa* M228 Is Required for Pathogenicity

**FIGURE 3:**
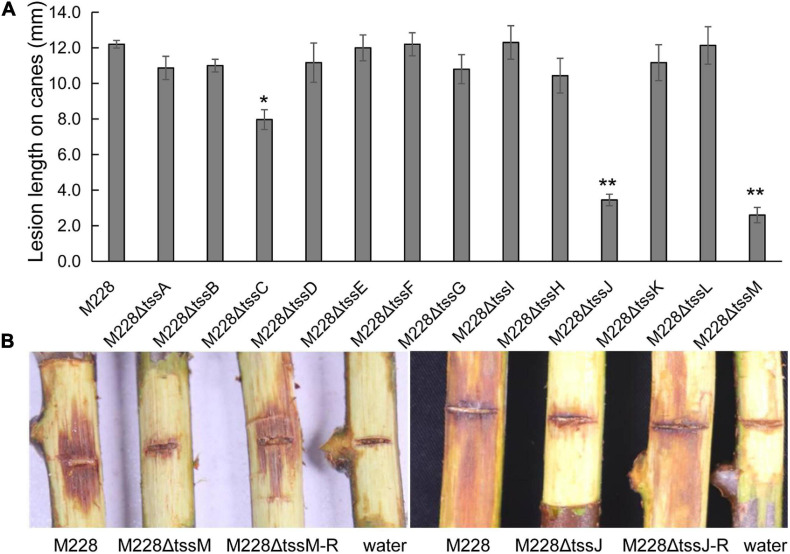
Pathogenicity analysis of the deletion mutant strains of 13 core genes constitute T6SS. **(A)** The lesion lengths analysis of the deletion mutants infecting on branches of kiwi. All the deletion mutants of 13 core genes of T6SS showed different degrees of pathogenicity reduction compared with that of the wild-type strain M228, especially gene *tssM* and *tssJ.* The pathogenicity of mutants M228ΔtssM and M228ΔtssJ mutants were significantly reduced by 78.7 and 71.3%, respectively. However, the complemented strain M228ΔtssM-R and M228ΔtssJ-R restored the pathogenicity to wild-type levels. The lesion lengths are the average of three replicate. Statistical significance was determined by Student’s *t*-test. “*” Means the difference is significant at the 0.05 level; “**” means the difference is significant at the 0.01 level. **(B)** The lesion observation of mutant strain M228ΔtssM and M228ΔtssJ infecting on branches of kiwi.

To clarify the role of T6SS in the pathogenicity of M228, the pathogenicity of mutants lacking the T6SS gene cluster or 13 core genes were assessed by conducting pathogenicity tests on healthy branches of kiwi (cv. “Hongyang”), as described previously (Zhao et al., 2019). The pathogenicity of the mutant M228ΔT6SS was reduced by 69.65% compared with that of the wild-type strain M228 (Figure 2A). All the deletion mutants of 13 core genes of T6SS showed different degrees of pathogenicity reduction compared with that of the wild-type strain M228 (Figure 3A), especially gene *tssM* and *tssJ* (Figure 3B). The pathogenicity of mutants M228ΔtssM and M228ΔtssJ mutants were significantly reduced by 78.7 and 71.3%, respectively. However, the complemented strain M228ΔtssM-R and M228ΔtssJ-R restored the pathogenicity to wild-type levels (Figure 3B).

**FIGURE 4:**
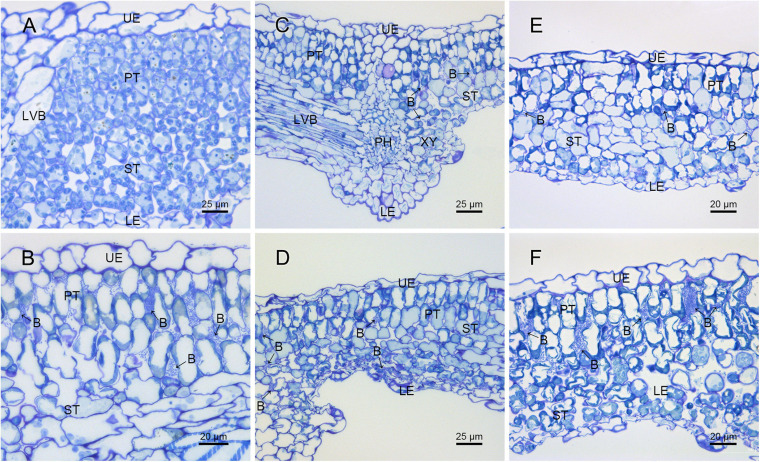
Histological observation of the pathogenic strain M228 and its mutants infecting leaves of kiwi by SEM. **(A)** Healthy kiwi leaves; **(B)** after 4 days of inoculation of the host kiwifruit leaves using wild-type strain M228, a large number of pathogens could be observed in the host tissue. **(C,E)** After 4 days of inoculation of the host kiwifruit leaves using the mutants M228ΔtssM and M228ΔtssJ, only a small amount of bacterial colonization was found in the host tissue; **(D,F)** after 4 days of inoculation of the host kiwifruit leaves using the mutantsM228ΔtssM-R and M228ΔtssJ-R, a large number of pathogens could be observed in the host tissue, and there was no significant difference in the colonization and infection ability between the wild-type strain M228 and the complemented strains. UE, upper epidermis; PT, palisade tissue; ST, sponge tissue; PH, phloem; XY, xylem; LE, lower epidermis; LVB, lateral vein; B, pathogenic bacteria.

**FIGURE 5:**
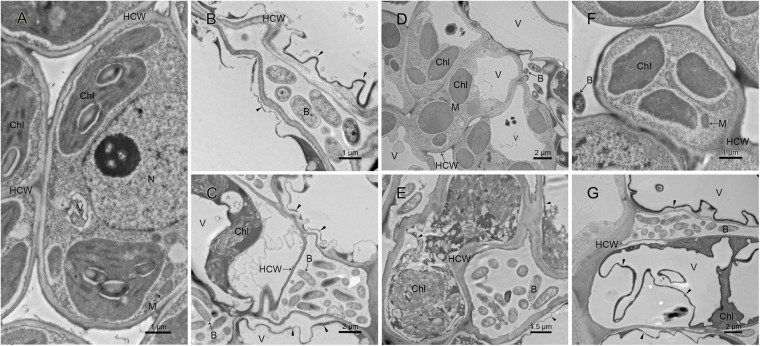
Ultrastructure analysis of kiwi leaves infected by the pathogenic strain M228 and its mutants by TEM. **(A)** Healthy kiwi leaves; **(B,C)** after 4 days of inoculation of the host kiwifruit leaves using wild-type strain M228, serious plasmolysis occurred in the host cells of the pathogenic bacteria colonized area, and electron density of the host cell wall decreased and its chloroplast was degraded; **(D,F)** after 4 days of inoculation of the host kiwifruit leaves using the mutants M228ΔtssM and M228ΔtssJ, the host cell was intact, and organelles were not significantly degraded due to pathogen colonization. **(E,G)** After 4 days of inoculation of the host kiwifruit leaves using the mutants M228ΔtssM-R and M228ΔtssJ-R, serious plasmolysis occurred in the host cells of the pathogenic bacteria colonized area, and electron density of the host cell wall decreased and its chloroplast was degraded; the mutants could cause a similar phenomenon to the host cell with wild-type strain M228. HCW, host cell wall; N, nucleus; V, vacuole; Chl, chloroplast; M, mitochondria; B, pathogenic bacteria; CW, bacterial cell wall; “▲,” host cell plasmolysis.

The difference in pathogenicity between M228 and the mutants was also confirmed by SEM (Figure 4) and TEM (Figure 5). After 4 days of inoculation of the host kiwifruit leaves using wild-type strain M228, a large number of pathogens could be observed in the host tissue (Figure 4B). Serious plasmolysis occurred in the host cells of the pathogenic bacteria colonized area, and electron density of the host cell wall decreased and its chloroplast was degraded (Figures 5B,C). After inoculation of the mutants M228ΔtssM (Figure 4C) and M228ΔtssJ (Figure 4E), only a small amount of bacterial colonization was found in the host tissue at 4 days. The host cell was intact, and the organelles were not significantly degraded due to pathogen colonization (Figures 5D,F). However, there was no significant difference in the colonization and infection ability between the wild-type strain M228 and the complemented strain M228ΔtssM-R (Figures 4D, 5E) and M228ΔtssJ-R (Figures 4F, 5G). The mutants can cause a similar phenomenon to the host cell, indicating that its infection ability is restored to the wild-type level. The results showed that the T6SS gene cluster played an important role in the pathogenicity of *Psa* in kiwifruit, especially the core genes *tssM* and *tssJ.*

### T6SS Gene Cluster Encodes a Functioning T6SS in *Psa* M228

**FIGURE 6:**
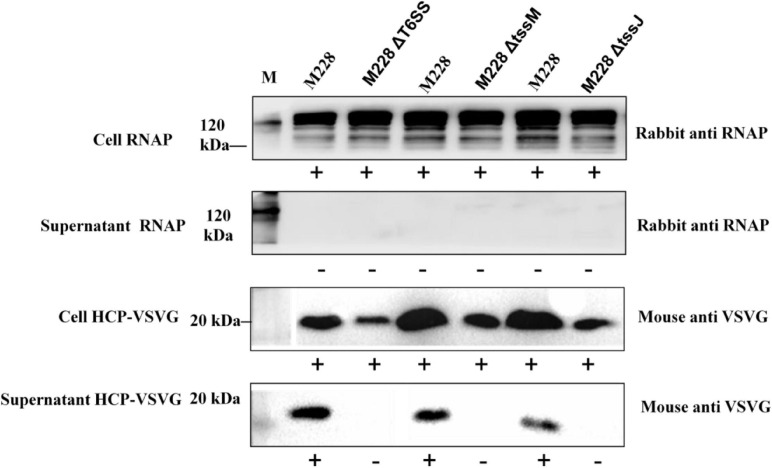
Immunoblot analysis of *Psa* M228 and mutants M228ΔT6SS, M228ΔtssM, and M228ΔtssJ Hcp export. Hcp was detected by western blot from both cells and supernatant for the wild-type strain Psa M228, but was only detected in the cells and not in the supernatant for mutant strain M228ΔT6SS, M228ΔtssM, and M228ΔtssJ. The internal control protein RNAP was stably expressed in the cell, but was not detected in the supernatant, confirming the reliability of the results. Lane M, protein marker.

To investigate the function of T6SS, M228 and the deletion mutant strain, M228ΔT6SS, M228ΔtssM, and M228ΔtssJ were examined for the secretion of Hcp, which is an indication of functional T6SS in many bacterial species (Ma and Mekalanos, 2010). As shown in Figure 6, Hcp was detected by western blot from both cells and supernatants for the wild-type strain *Psa* M228, but only detected in the cells and not in the supernatant for mutant strains M228ΔT6SS, M228ΔtssM, and M228ΔtssJ. This result demonstrated that T6SS is essential for the secretion of Hcp in *Psa* M228. The results also showed that T6SS in the pathogenic strain *Psa*M228 is functional, and its core genes *tssM* and *tssJ* are critical for Hcp secretion and pathogenicity. The internal control protein RNAP was stably expressed in the cell, but was not detected in the supernatant, confirming the reliability of the results.

### The T6SS of *Psa* M228 Is Used to Compete Against Bacteria

**FIGURE 7:**
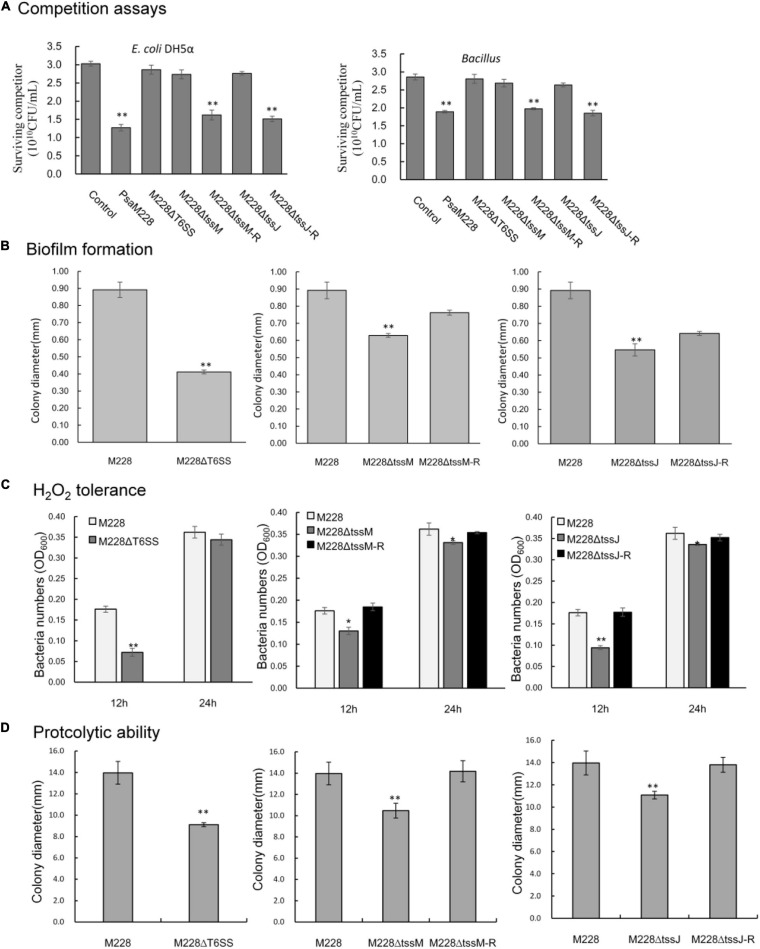
Competitive, biofilm formation, and environmental adaptability analysis of wild-type strain M228 and mutants M228ΔT6SS, M228ΔtssM, and M228ΔtssJ. **(A)** Bacterial competition assay. When the competitor strains were cocultured with wild-type *Psa* M228 or the complementation mutants M228ΔtssM-R and M228ΔtssJ-R, the viable cell numbers showed an obvious drop from 46.5, 58.0 to 31.0, and 35.2%, respectively. In contrast, when the competitor strains were cocultured with deletion mutants M228ΔT6SS, M228ΔtssM, and M228ΔtssJ, the survival of competitor bacteria increased up to the same level as that of the control. **(B)** Biofilm assays. The biofilm formation ability of the three mutants M228ΔT6SS, M228ΔtssM, and M228ΔtssJ was significantly lower than that of the wild-type strain M228 and decreased by 53.8, 29.5, and 38.7%, respectively. The biofilm formation ability was restored to the same level as that of M228 when the deletion genes were restored. **(C)** H_2_O_2_ tolerance assay. The H_2_O_2_ tolerance of mutant strains M228ΔT6SS, M228ΔtssM, and M228ΔtssJ was significantly lower than that of the wild-type strain M228, decreased by 59.1, 26.1, and 46.6% at 12 h, respectively, and decreased by 5.0, 8.5, and 7.2% at 24 h, respectively. The H_2_O_2_ tolerance was restored when the deletion genes were restored to the same level as that of M228 when the deletion genes were restored. **(D)** Proteolytic ability assay. The proteolytic ability of mutant strains M228ΔT6SS, M228ΔtssM, and M228ΔtssJ was significantly lower than that of the wild-type strain M228, and decreased by 34.8, 25.0, and 20.66%, respectively. The proteolytic ability was restored when the deletion genes were restored to the same level as that of M228. Statistical significance was determined by Student’s *t*-test. “*” Means the difference is significant at the 0.05 level; “**” means the difference is significant at the 0.01 level.

In order to determine whether the T6SS is functional in competition between bacterial species, a competitive test was conducted in order to examine whether the mutants lacking the T6SS gene clusters could reduce the number of *E. coli* DH5α and *Bacillus* strains on agar plates. When the competitor strains were cocultured with wild-type *Psa* M228 or the complementation mutants M228ΔtssM-R and M228ΔtssJ-R, viable cell numbers showed an obvious drop from 46.5, 58.0 to 31.0, and 35.2%, respectively. In contrast, when the competitor strains were cocultured with deletion mutants M228ΔT6SS, M228ΔtssM, and M228ΔtssJ, the survival of competitor bacteria was increased up to the same level as that of the control (Figure 7A). Overall, the results showed that the T6SS gene cluster is related to bacterial competition in *Psa* M228.

### T6SS of *Psa* M228 Is Required for Biofilm Formation

To investigate the function of T6SS, M228, and the deletion mutant strains, M228ΔT6SS, M228ΔtssM, and M228ΔtssJ were examined for biofilm formation. As shown in Figure 7B, the biofilm formation ability of the three mutants was significantly lower than that of the wild-type strain M228 (*P* < 0.05): M228ΔT6SS decreased by 53.8%, M228ΔtssM by 29.5%, and M228ΔtssJ by 38.7%. After the deletion genes were restored, the biofilm formation ability of the mutants was restored to the same level as that of M228.

### The T6SS of *Psa* M228 Is Required for H_2_O_2_ Tolerance and Proteolytic Ability

In order to determine whether the T6SS is functional in environmental adaptability of *Psa* M228, we examined whether the mutants lacking the T6SS gene clusters could weaken the H_2_O_2_ tolerance and proteolytic ability.

The H_2_O_2_ tolerance results of mutants M228ΔT6SS, M228ΔtssM, and M228ΔtssJ was significantly lower than that of the wild-type strain M228, decreased by 59.1, 26.1, and 46.6% at 12 h, respectively, and decreased by 5.0, 8.5, and 7.2% at 24 h, respectively (Figure 7C). The hydrogen peroxide tolerance was significantly affected by T6SS in the early stages. The proteolytic ability of mutants M228ΔT6SS, M228ΔtssM, and M228ΔtssJ was significantly lower than that of the wild-type strain M228 (*P* < 0.01), and decreased by 34.8, 25.0, and 20.66%, respectively (Figure 7D). The H_2_O_2_ tolerance and proteolytic ability were all restored when the deletion genes were restored.

### T3SS-Related Genes Show Down-Regulated Expression in *Psa* M228ΔT6SS

**FIGURE 8:**
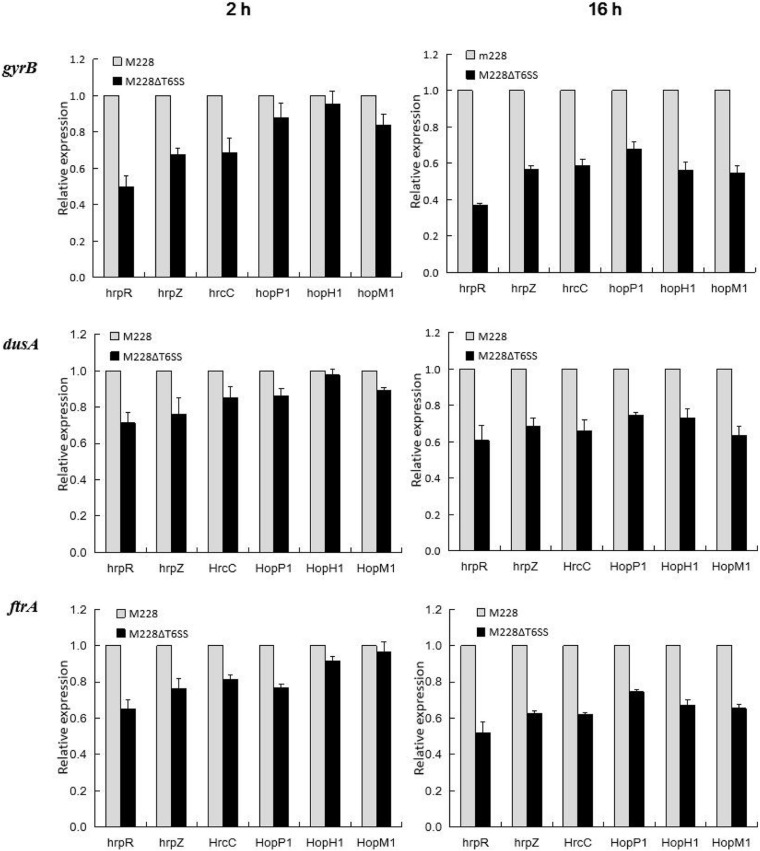
Differential expression of T3SS-related genes analysis in the process of pathogenic strain *Psa* M228 and its mutant M228ΔT6SS infecting kiwi branches. During the interaction between the mutant strain M228ΔT6SS and the host, the expression levels of the T3SS transcriptional regulatory genes *hrpR*, structural genes *hrpZ*, *hrcC*, *hopP1*, and effector genes *hopM1* and *hopH1*were regulated. Following interaction for 2 h, T3SS-related genes *hrpR*, *hrpZ*, *hrcC*, *hopP1*, *hopH1*, and *hopM1* of the mutant M228ΔT6SS, were down-regulated by 2.01, 1.48, 1.46, 1.14, 1.05, and 1.2 times compared to the wild-type strain; following interaction for 16 h, the expression of the genes was down-regulated by 2.71, 1.76, 1.70, 1.48, 1.78, and 1.83 times, using *gyrB* as internal reference; a similar downregulation result was obtained using *dusA* and *ftrA* as internal reference genes.

The qRT-PCR technique was used to analyze the relationship between the T6SS cluster gene and T3SS-related gene expression. During the interaction between the mutant strain M228ΔT6SS and the host, expression levels of T3SS transcriptional regulatory genes *hrpR*, structural genes *hrpZ*, *hrcC*, *hopP1*, and effector genes *hopM1* and *hopH1*were regulated. Following interaction for 2 h, T3SS-related genes *hrpR*, *hrpZ*, *hrcC*, *hopP1*, *hopH1*, and *hopM1* of the mutant M228ΔT6SS, were down-regulated by 2.01, 1.48, 1.46, 1.14, 1.05, and 1.2 times compared to the wild-type strain; following interaction for 16 h, the expression of the genes was down-regulated by 2.71, 1.76, 1.70, 1.48, 1.78, and 1.83 times, using *gyrB* as an internal reference. A similar down-regulation result was obtained using *dusA* and *ftrA* as internal reference genes (Figure 8). The expression levels of the T3SS related genes were significantly affected by T6SS in the later stage.

## Discussion

The protein secretion system plays an important role in the pathogenicity and host infection process of many bacterial pathogens (Gerlach and Hensel, 2007). Among them, T3SS plays a vital role in the pathogenicity of many pathogenic bacteria and can directly transfer effectors from pathogenic bacteria to host cells and exerts pathogenic functions (Cornelis et al., 2006; Zhang et al., 2018). Many studies have confirmed that T3SS plays an important role in the phytopathogenic bacteria of a variety of economic crops. Our previous studies also indicated that T3SS promoters are important pathogenic factors (Zhao et al., 2019) by the differential proteome data of strong and weak pathogenic strains. However, in this study, a T6SS cluster consisting of 13 core genes (A-J) was found in *Psa* M228. Although there is a frameshift mutation in the transmembrane structural protein gene *tssM* (1272 codons) and the ATP hydrolase gene *tssH* (854 codons), the pathogenicity was weaker in all deletion mutants, including mutants M228ΔtssM and M228ΔtssH, than in the wild-type strain M228. It seems that the frameshift mutations of these two genes did not affect their function. This mechanism requires further research.

We further explored whether the gene cluster in *Psa* 228 is a functional T6SS and plays a similar function in pathogenicity as it does in other pathogenic bacteria. Western blot results showed that the T6SS was necessary for Hcp secretion, showing that the T6SS gene cluster in M228 is functional. To determine whether the observed decrease in pathogenicity is related to a functioning T6SS, we generated 13 core genes of T6SS gene cluster deletion mutants. These mutations all led to attenuated pathogenesis and weakened the spread ability of kiwi plants at different levels, especially *tssM* and *tssJ.* All these properties of the two gene complementation mutants were restored to the same level as those of the wild-type strain M228. The results indicate that the pathogenicity reduction is related to T6SS. To find out whether the effect of T6SS on pathogenicity is related to T3SS, we also conducted a preliminary study. Quantitative real-time results showed that T3SS genes related to pathogenicity were all down regulated in mutant M228ΔT6SS compared to those in the wild-type strain *Psa* M228, especially in the later stages of infection. Further research is needed to understand whether T6SS affects pathogenicity by regulating the T3SS-related genes expressed indirectly or by secreting virulence factors directly. The study results are consistent with the experimental results of a single T6SS gene mutation in *R. solanacearum* (Luo et al., 2016) and the relationship between T3SS and T6SS in the pathogenicity of *Edwardsiella tarda* (Zhang et al., 2016).

In nature or in the host, microorganisms usually exist in complex environmental conditions. For their own survival, they must compete with other organisms for limited nutrients and space. Studies have shown that many Gram-negative bacteria can kill or inhibit other bacteria through T6SS (Alcoforado et al., 2015). For example, the human pathogen *Vibrio cholerae* kills *E. coli* by secreting virulence factors through T6SS (MacIntyre et al., 2010). In addition, recent studies have shown that T6SS plays an important role in *P. aeruginosa* and *E. coli* cells. A variety of antibacterial toxins that plays an important role in bacterial competition can be delivered to the target cell through T6SS (Basler et al., 2013; Brunet et al., 2013; Durand et al., 2014; Alcoforado et al., 2015). It has been reported that *E. coli* is susceptible to T6SS and can be killed by the antibacterial toxin secreted by T6SS. Therefore, *E. coli* is often used as an ideal competitor (MacIntyre et al., 2010; Zheng et al., 2011; Weber et al., 2013). *Bacillus* is one of the most abundant species of plant endophytes (Gadhave et al., 2018; Gordon et al., 2020). Therefore, in this work, we used *E. coli* and *Bacillus* as competitors. Our results showed that T6SS is required for efficient bacterial competition. Moreover, how the competition is carried out *in vitro* requires further research and needs to be confirmed using *in vivo* survival assays. In addition, we investigated the role of the T6SS in bacterial biofilm formation, which is essential during infection and pathogenic processes (Yu et al., 2016). Our research indicated that T6SS plays an important role in the pathogenicity of *Psa* M228. These results present novel insights regarding the pathogenesis of *Psa*.

The adaptability of plants to various pressures from the natural environment needs to be achieved by regulating the pressure signal transduction pathways. When plants are attacked by pathogenic bacteria, it produces a series of stress responses, including the oxidative burst (Nicaise and Candresse, 2017; Kanwar and Jha, 2019; Guo and Li, 2000). Previous studies have suggested that these reactive oxygen species have pathological functions such as inhibiting the growth of bacterial and fungal pathogens (Jiang et al., 2011). Our study showed that compared to the wild-type strain M228, the growth of the deletion mutants M228ΔT6SS, M228ΔtssM, and M228ΔtssJ was significantly inhibited under H_2_O_2_ stress. The deletion of T6SS, *tssM*, and *tssJ* from *Psa* M228 may lead to reduced tolerance to H_2_O_2_, thereby affecting the survival of the pathogenic bacteria. The results indicate that T6SS may be associated with resistance to environmental stress in pathogenic bacteria. However, this requires further investigation.

## Conclusion

Here, we found a T6SS gene cluster consisting of 13 core genes (A-M) in the genome of *Psa* M228 based on a genome-wide analysis. We provide evidence that T6SS plays an important role in pathogenesis, bacterial competition, biofilm formation, and environmental adaptability. The next step is to clarify the assembly and secretion mechanism of T6SS and identify the virulence factors secreted by T6SS in *Psa*. This research has deepened our understanding of the pathogenic mechanism of *Psa*.

## Data Availability Statement

The raw data supporting the conclusions of this article will be made available by the authors, without undue reservation.

## Author Contributions

NW, NH, and LH designed the research. NW, NH, RT, XG, JC, ZW, and YL performed the experiments. NW, NH, RT, ZW, and YL analyzed the data. NW and NH wrote the manuscript. All authors contributed to the article and approved the submitted version.

## Conflict of Interest

The authors declare that the research was conducted in the absence of any commercial or financial relationships that could be construed as a potential conflict of interest.

## Footnotes

1 ftp://ftp.ncbi.nih.gov/pub/COG/COG2014/static/lists/listAciave.html

## References

[B1] Agnetti J. Seth-Smith H. M. B. Ursich S. Reist J. Basler M. Nickel C. (2019). Clinical impact of the type VI secretion system on virulence of *Campylobacter* species during infection. *BMC Infect. Dis.* 19:237. 10.1186/s12879-019-3858-x 30845966 PMC6407262

[B2] Alcoforado D. J. Liu Y. C. Coulthurst S. J. (2015). Molecular weaponry: Diverse effectors delivered by the Type VI secretion system. *Cell Microbiol.* 17 1742–1751. 10.1111/cmi.12532 26432982 PMC4832377

[B3] Asolkar T. Ramesh R. (2020). The involvement of the type six secretion system (T6SS) in the virulence of *Ralstonia solanacearum* on brinjal. 3. *Biotech* 10:324. 10.1007/s13205-020-02311-4 32656057 PMC7324442

[B4] Basler M. Ho B. T. Mekalanos J. J. (2013). Tit-for-tat: Type VI secretion system counterattack during bacterial cell-cell interactions. *Cell* 152 884–894. 10.1016/j.cell.2013.01.042 23415234 PMC3616380

[B5] Ben-aakov R. Salomon D. (2019). The regulatory network of *Vibrio parahaemolyticus* type VI secretion system. *Environ. Microbiol.* 21 2248–2260. 10.1111/1462-2920.14594 30882997 PMC6618117

[B6] Broms J. E. Lavander M. Sjo-stedt A. (2009). A conserved a-helix essential for a type VI secretion-like system of *Francisella tularensis*. *J. Bacteriol.* 191 2431–2446. 10.1128/jb.01759-08 19201795 PMC2668417

[B7] Brunet Y. R. Espinosa L. Harchouni S. Mignot T. Cascales E. (2013). Imaging type VI secretion-mediated bacterial killing. *Cell Rep.* 3 36–41. 10.1016/j.celrep.2012.11.027 23291094

[B8] Catarina F. R. Eric D. Marie-Stéphanie A. Stéphanie B. Miguel O. L. Badreddine D. (2011). Towards a structural comprehension of bacterial type VI secretion systems: characterization of the TssJ-TssM complex of an *Escherichia coli* pathovar. *PLoS Pathog.* 7:e1002386. 10.1371/journal.ppat.1002386 22102820 PMC3213119

[B9] Cianfanelli F. R. Monlezun L. Coulthurst S. J. (2016). Aim, load, fire: the Type VI secretion system, a bacterial nanoweapon. *Trends Microbiol.* 24 51–62. 10.1016/j.tim.2015.10.005 26549582

[B10] Cornelis G. R. Agrain C. Sorg I. (2006). Length control of extended protein structures in bacteria and bacteriophages. *Curr. Opin. Microbiol.* 9 201–206. 10.1016/j.mib.2006.01.002 16458574

[B11] Costa T. R. D. Felisberto-Rodrigues C. Meir A. Prevost M. S. Redzej A. Trokter M. (2015). Secretion systems in gram-negative bacteria: Structural and mechanistic insights. *Nat. Rev. Microbiol.* 13 343–359. 10.1038/nrmicro3456 25978706

[B12] Deb S. Gupta M. K. Patel H. K. Sonti R. V. (2019). *Xanthomonasoryzae* pv. *oryzaexopq* protein suppresses rice immune responses through interaction with two 14-3-3 proteins but its phosphor-null mutant induces rice immune responses and interacts with another 14-3-3 protein. *Mol. Plant Patholmol. Plant Pathol.* 20 976–989. 10.1111/mpp.12807 31094082 PMC6856769

[B13] Donati I. Cellini A. Sangiorgio D. Vanneste J. L. Spinelli F. (2020). *Pseudomonas syringae* pv. *actinidiae*: ecology, infection dynamics and disease epidemiology. *Microb. Ecol.* 80 1–22. 10.1007/s00248-019-01459-8 31897570 PMC7223186

[B14] Durand E. Cambillau C. Cascales E. Journet L. (2014). VgrG, Tae, Tle, and beyond: The versatile arsenal of type VI secretion effectors. *Trends Microbiol.* 22 498–507. 10.1016/j.tim.2014.06.004 25042941

[B15] Felsenstein J. (1985). Confidence limits on phylogenies: An approach using the bootstrap. *Evolution* 39 783–791. 10.1111/j.1558-5646.1985.tb00420.x 28561359

[B16] Gadhave K. R. Devlin P. F. Ebertz A. Ross A. Gange A. C. (2018). Soil inoculation with *Bacillus* spp. modifies root endophytic bacterial diversity, evenness, and community composition in a context-specific manner. *Microb. Ecol.* 76 741–750. 10.1007/s00248-018-1160-x 29511840 PMC6132550

[B17] Gao X. Huang Q. Zhao Z. Han Q. Ke X. Qin H. (2016). Studies on the infection, colonization, and movement of *Pseudomonas syringae* pv. *Actinidiae* in kiwifruit tissues using a GFPuv-Labeled strain. *PLoS One* 11:e0151169. 10.1371/journal.pone.0151169 26999596 PMC4801369

[B18] Gerlach R. G. Hensel M. (2007). Protein secretion systems and adhesins: The molecular armory of gram-negative pathogens. *Int. J. Med. Micobiol.* 297 401–415. 10.1016/j.ijmm.2007.03.017 17482513

[B19] Gordon W. Mullins A. J. Edward C. O. Arun R. Bai A. J. Eshwar M. (2020). Culturable diversity of bacterial endophytes associated with medicinal plants of the Western Ghats, india. *FEMS Microbiol. Ecol.* 9:147. 10.1093/femsec/fiaa147 32710748 PMC7422900

[B20] Guo Z. Li D. (2000). Active oxygen species in plant disease resistance. *J. Integr. Plant Biol.* 42 881–891.

[B21] Habbadi K. Benkirane R. Benbouazza A. Bouaichi A. Achbani E. H. (2017). Biological control of grapevine crown gall caused by allorhizobiumvitis using bacterial antagonists. *IJSR* 6 1390–1397. 10.21275/ART20174478

[B22] Hang C. Yue H. Qin K. Yang X. Jia Z. Li Q. (2018). A serological approach for the identification of the effector hopz5 of *Pseudomonas syringae* pv. *actinidiae*: a tool for the rapid immune detection of kiwifruit bacterial canker. *J. Plant Pathol.* 100 171–177. 10.1007/s42161-018-0041-y

[B23] Hirose K. Ishiga Y. Fujikawa T. (2020). Phytotoxin synthesis genes and type III effector genes of *Pseudomonas syringae* pv. *Actinidiae* biovar 6 are regulated by culture conditions. *PEERJ* 8:e9697. 10.7717/peerj.9697 32864217 PMC7430302

[B24] Huang Q. L. Gao X. N. Zhao Z. B. Qin H. Q. Huang L. L. (2013). Transformed GFPuv into *Pseudomonas syringae* pv. *actinidiae* and its biological characteristics and colonization in soil and roots of kiwifruit. *Scient. Agricult. Sin.* 46 282–291.

[B25] Jiang S. J. Liu Z. Yu H. Zhang G. Q. Fan W. Y. (2011). Research on oxidative stress Induced by Tenuazonic Acid from Alternaria augustiovoide and Changes in Antioxidant enzyme activities in leaves of Echinochloacrus-gall. *J. Agr. Sci. Tech. Iran* 2011 792–798. 10.16175/j.cnki.1009-4229.2011.06.019

[B26] Kang Z. S. (1995). *Ultrastructure of plant pathogenic fungi.* Beijing: China Science and Technology Press.

[B27] Kanwar P. Jha G. (2019). Alterations in plant sugar metabolism: signatory of pathogen attack. *Planta* 249 305–318. 10.1007/s00425-018-3018-3 30267150

[B28] Kapitein N. Bonemann G. Pietrosiuk A. Seyffer F. € Hausser I. Locker J. K. (2013). ClpV recycles VipA/VipB tubules and prevents non-productive tubule formation to ensure efficient type VI protein secretion. *Mol. Microbiol.* 87 1013–1028. 10.1111/mmi.12147 23289512

[B29] Khan M. Seto D. Subramaniam R. Desveaux D. (2018). Oh, the places they’ll go! A survey of phytopathogen effectors and their host targets. *Plant J. Cell Mol. Biol.* 93 651–663. 10.1111/tpj.13780 29160935

[B30] Kim M. J. Chae D. H. Cho G. Kim D. R. Kwak Y. S. (2019). Characterization of antibacterial strains against kiwifruit bacterial canker pathogen. *Plant Pathol. J.* 35 1–13. 10.5423/PPJ.OA.05.2019.0154 31632222 PMC6788412

[B31] Kudryashev M. Wang R. Y.-R. Brackmann M. Scherer S. Maier T. Baker D. (2015). Structure of the type VI secretion system contractile sheath. *Cell* 160 952–962. 10.1016/j.cell.2015.01.037 25723169 PMC4359589

[B32] Kvitko B. H. Collmer A. (2011). Construction of *Pseudomonas syringae*pv. *Tomato* DC3000 mutant and polymutant strains. *Methods Mol. Biol.* 712:109. 10.1007/978-1-61737-998-7_10 21359804

[B33] Le S. Q. Gascuel O. (1993). An improved general amino acid replacement matrix. *Mol. Biol. Evol.* 25 1307–1320. 10.1093/molbev/msn067 18367465

[B34] Leiman P. G. Basler M. Ramagopal U. A. Bonanno J. B. Sauder J. M. Pukatzki S. (2009). Type VI secretion apparatus and phage tail-associated protein complexes share a common evolutionary origin. *Proc. Natl. Acad. Sci. U S A.* 106 4154–4159. 10.1073/pnas.0813360106 19251641 PMC2657435

[B35] Lopez I. Pardo M. A. (2005). Application of relative quantification taqman real-time polymerase chain reaction technology for the identification and quantification of *Thunnus alalunga* and *Thunnus albacares*. *J. Agr. Food Chem.* 53 4554–4560. 10.1021/jf0500841 15913324

[B36] Luo Y. Xu J. Xu J. Zhang H. Feng J. (2016). Functional analysis of a T6SS cluster of *Ralstonia solanacearum* po82. *Plant Prot. Sci.* 6 38–45.

[B37] Ma A. T. Mekalanos J. J. (2010). In vivo actin cross-linking induced by *Vibrio cholerae* type VI secretion system is associated with intestinal inflammation. *Proc. Natl. Acad. Sci U S A.* 107 4365–4370. 10.1073/pnas.0915156107 20150509 PMC2840160

[B38] MacIntyre D. L. Miyata S. T. Kitaoka M. Pukatzki S. (2010). The *Vibrio cholerae* type VI secretion system displays antimicrobial properties. *Proc. Natl. Acad. Sci. U S A.* 107 19520–19524. 10.1073/pnas.1012931107 20974937 PMC2984155

[B39] Mathias G. Victorien D. Corinne B. Thibaut R. Annabelle M. (2017). Contribution of the *Pseudomonas fluorescens* mfe01 type VI secretion system to biofilm formation. *PLoS One* 12:e0170770. 10.1371/journal.pone.0170770 28114423 PMC5256989

[B40] Mattinen L. Nissinen R. Riipi T. (2010). Host-extract induced changes in the secretome of the plant pathogenic bacterium *Pectobacterium atrosepticum*. *Proteomics* 7 3527–3537. 10.1002/pmic.200600759 17726675

[B41] Mcquade R. Stock S. (2018). Secretion systems and secreted proteins in gram-negative entomopathogenic bacteria: their roles in insect virulence and beyond. *Insects* 9:68. 10.3390/insects9020068 29921761 PMC6023292

[B42] Michelotti V. Lamontanara A. Burian G. Orrù L. Cellini A. Donati I. (2018). Comparative transcriptome analysis of the interaction between *Actinidiachinensis* var. *chinensis* and *Pseudomonas syringae*pv. *actinidiae* in absence and presence of acibenzolar-s-methyl. *BMC Genomics* 19:585. 10.1186/s12864-018-4967-4 30081820 PMC6090863

[B43] Molina L. Rezzonico F. Défago G. Duffy B. (2005). Autoinduction in erwinia amylovora: evidence of an acyl-homoserine lactone signal in the fire blight pathogen. *J. Bacteriol.* 187 3206–3213. 10.1128/JB.187.9.3206-3213.2005 15838048 PMC1082838

[B44] Nguyen V. S. Douzi B. Durand E. Roussel A. Cascales E. Cambillau C. (2018). Towards a complete structural deciphering of type VI secretion system. *Curr. Opin. Struc. Biol.* 49 77–84. 10.1016/j.sbi.2018.01.007 29414515

[B45] Nicaise V. Candresse T. (2017). Plum pox virus capsid protein suppresses plant pathogen-associated molecular pattern (pamp)-triggered immunity. *Mol. Plant Pathol.* 18 878–886. 10.1111/mpp.12447 27301551 PMC6638313

[B46] Pell L. G. Kanelis V. Donaldson L. W. Howell P. L. Davidson A. R. (2009). The phage lambda major tail protein structure reveals a common evolution for long-tailed phages and the type VI bacterial secretion system. *Proc. Natl. Acad. Sci. U S A.* 106 4160–4165. 10.1073/pnas.0900044106 19251647 PMC2657425

[B47] Pinheiro L. A. M. Pereira C. Barreal M. E. Gallego P. P. Almeida A. (2020). Environmental biotechnology use of phage ϕ6 to inactivate *Pseudomonas syringae* pv. *actinidiae* in kiwifruit plants: in vitro and ex vivo experiments. *Appl. Microbiol. Biot.* 104 1319–1330. 10.1007/s00253-019-10301-7 31853568

[B48] Pukatzki S. McAuley S. B. Miyata S. T. (2009). The type VI secretion system: translocation of effectors and effector-domains. *Curr. Opin. Microbiol.* 12 11–17. 10.1016/j.mib.2008.11.010 19162533

[B49] Sana T. G. Benjamin B. Sophie B. (2016). The T6SS of *Pseudomonas aeruginosa* strain PAO1 and their effectors: beyond bacterial-cell targeting. *Front. Cell Infect. Microbiol.* 6:61. 10.3389/fcimb.2016.00061 27376031 PMC4899435

[B50] Schafer A. Tauch A. Jager W. Kalinowski J. Thierbach G. Puhler (1994). A Small mobilizable multi-purpose cloning vectors derived from the *Escherichia coli* plasmids pK18 and pK19: selection of defined deletions in the chromosome of *Coryne bacterium glutamicum*. *Gene* 145 69–73. 10.1016/0378-1119(94)90324-7 8045426

[B51] Shyntum D. Y. Theron J. Moleleki L. N. Toth I. K. Coutinho T. A. (2015). *Pantoeaananatis* utilizes a type VI secretion system for pathogenesis and bacterial competition. *Mol. Plant Microbe Interact.* 28 420–431. 10.1094/MPMI-07-14-0219-R 25411959

[B52] Stefani E. Giovanardi D. (2011). Dissemination of *Pseudomonas syringae* pv. *actinidiae* through pollen and its epiphytic life on leaves and fruits. *Phytopathol. Mediterr.* 50 489–496. 10.1016/j.phymed.2011.09.068

[B53] Stepanovic S. Vukovic D. Dakic I. Savic B. Svabic-Vlahovic M. (2000). A modified microtiter-plate test for quantification of staphylococcal biofilm formation. *J. Microbiol. Meth.* 40 175–179. 10.1016/S0167-7012(00)00122-6 10699673

[B54] Tamura K. Stecher G. Peterson D. Filipski A. Kumar S. (2013). MEGA6: Molecular evolutionary genetics analysis version 6.0. *Mol. Biol. Evol.* 30 2725–2729. 10.1093/molbev/mst197 24132122 PMC3840312

[B55] Tontou R. Giovanardi D. Stefani E. (2014). Pollen as a possible pathway for the dissemination of *Pseudomonas syringae* pv. *actinidiae* and bacterial canker of kiwifruit. *Phytopathol. Mediterr.* 53 333–339. 10.14601/Phytopathol_Mediterr-13757

[B56] Vanneste J. L. (2017). The scientific, economic, and social impacts of the New Zealand outbreak of bacterial canker of kiwifruit (*Pseudomonas syringae* pv. *actinidiae*). *Annu. Rev. Phytopathol.* 55 377–399. 10.1146/annurev-phyto-080516-035530 28613977

[B57] Weber B. S. Miyata S. T. Iwashkiw J. A. Mortensen B. L. Pukatzki S. Feldman M. F. (2013). Genomic and functional analysisof the type VI secretion system in *Acinetobacter*. *PLoS One* 8:e55142. 10.1371/journal.pone.0055142 23365692 PMC3554697

[B58] Wen Z. Yan G. Qian C. Qi Z. Lin F. Zheng J. (2018). Decorin is a pivotal effector in the extracellular matrix and tumour microenvironment. *Oncotarget* 9 5480–5491. 10.18632/oncotarget.23869 29435195 PMC5797066

[B59] Williams H. Ting C. Nejati M. Jones M. H. Penhall N. Lim J. Y. (2020). Improvements to and large-scale evaluation of a robotic kiwifruit harvester. *J. Robotic Syst.* 37 187–201. 10.1002/rob.21890

[B60] Wu C. F. Smith D. A. Lai E. M. Chang J. H. (2018). The agrobacterium type VI secretion system: a contractile nanomachine for interbacterial competition. *Curr. Top Microbiol.* 418 215–231. 10.1007/82_2018_99 29992360

[B61] Yu Q. Li J. Zhang Y. Wang Y. Liu L. Li M. (2016). Inhibition of gold nanoparticles (aunps) on pathogenic biofilm formation and invasion to host cells. *Sci Rep. U K.* 6:26667. 10.1038/srep26667 27220400 PMC4879543

[B62] Zhang L. Ni C. Xu W. Dai T. Liu Q. (2016). Intra macrophage infection reinforces the virulence of *Edward siellatarda*. *J. Bacteriol.* 198 1534–1542. 10.1128/JB.00978-15 26953340 PMC4859603

[B63] Zhang L. Xu J. Xu J. Chen K. He L. Feng J. (2012). TssM is essential for virulence and required for type VI secretion in *Ralstonia solanacearum*. *J. Plant Dis. Protect.* 119 125–134. 10.1007/BF03356431

[B64] Zhang L. Xu J. Xu J. Zhang H. He L. Feng J. (2014). TssB is essential for virulence and required for Type VI secretion system in *Ralstonia solanacearum*. *Microb. Pathog.* 74 1–7. 10.1016/j.micpath.2014.06.006 24972114

[B65] Zhang Y. Liu Y. Wang T. Deng X. Chu X. (2018). Natural compound sanguinarine chloride targets the type III secretion system of *Salmonella entericasero* var *typhimurium*. *Biochem. Biophys. Rep.* 14 149–154. 10.1016/j.bbrep.2018.04.011 29761161 PMC5948472

[B66] Zhao Z. Chen J. Gao X. Zhang D. Zhang J. Wen J. (2019). Comparative genomics reveal pathogenicity-related loci in *Pseudomonas syringae* pv. A*ctinidiae* biovar 3. *Mol. Plant Pathol.* 20 923–942. 10.1111/mpp.12803 31025813 PMC6589868

[B67] Zheng J. Ho B. Mekalanos J. J. (2011). Genetic analysis of antiamoebae and anti-bacterial activities of the type VI secretion system in *Vibriocholerae*. *PLoS One* 6:e23876. 10.1371/journal.pone.0023876 21909372 PMC3166118

[B68] Zhu P. C. Li Y. M. Zou H. F. Niu X. N. Xu L. H. Jiang W. (2020). Type VI secretion system is not required for virulence on rice but for inter-bacterial competition in *Xanthomonasoryzae* pv. *Oryzicola*. *Res. Microbiol.* 171 64–73. 10.1016/j.resmic.2019.10.004 31676435

