# Supplementary material for: Role of the Type VI Secretion System in the Pathogenicity of Pseudomonas syringae pv. actinidiae, the Causative Agent of Kiwifruit Bacterial Canker
Source: Front Microbiol. 2021 Feb 19;12:627785. doi: 10.3389/fmicb.2021.627785 (PMC7933208; doi:10.3389/fmicb.2021.627785)
Supplement: Supplementary file 1 [file Table_1.doc]

**Table S1. PCR primers used in this study**

| **Primer pairs** | **Primer Sequence(5’-3’)** | **Product size(bp)** | **Gene** | **Reference** |
| --- | --- | --- | --- | --- |
| Psa-F  Psa-R | CAGAGGCGCTAACGAGGAAA  CGAGCATACATCAACAGGTCA | 311 | *hopZ3* | Balestra et al.2013 |
| T6SS-Q-F  T6SS-Q-R | CTATGACATGATTACGAATTCTTTGGGGCGGTTGAATGAGA  GGAACGCAGCTACGTATGGACCAATCCCGTTATCGTCGGT | 1790 | Upstream T6SS cluster | This study |
| T6SS-H-F  T6SS-H-R | TCCATACGTAGCTGCGTTCC  ACGACGGCCAGTGCCAAGCTTAAGCCGTTGCATTCTCGGTA | 2540 | Dowm stream T6SS cluster | This study |
| SacB-F  SacB-R | GCAAACACTGGAACTGAAGATGG  TTCCTTTCGCTTGAGGTACAGC | 478 | *SacB* | This study |
| TssA-F  TssA-R | TGACCCGCAAGCAGGTAC  AGCGATGCACAACAAATGG | 449 | *tssA* | This study |
| TssA-Q-F  TssA-Q-R | CTATGACATGATTACGAATTCGGCTGGGGGGCTGCTGATAC  GAACTGACCCGGGCGGATAG | 614 | In-frame deletion of *tssA* | This study |
| TssA-H-F  TssA-H-R | CTATCCGCCCGGGTCAGTTCTTGCTGGGCGTGCTGTAAG  ACGACGGCCAGTGCCAAGCTTCTGACTGGTGATGCCGTGAG | 798 | In-frame deletion of *tssA* | This study |
| TssB-F  TssB-R | CCGATCGACCGACGCTTTGT  GCTTGTCCAGGTCGTCGTT | 268 | *tssB* | This study |
| TssB-Q-F  TssB-Q-R | CTATGACATGATTACGAATTCCAACACGAATGCTCAACGGC  GTGAACGACGACGATCAGGCCATGGTGGCTGAACTCCGAT | 992 | In-frame deletion of *tssB* | This study |
| TssB-H-F  TssB-H-R | GCCTGATCGTCGTCGTTCAC  ACGACGGCCAGTGCCAAGCTTTGCCCCACAGGTATTTGCTA | 879 | In-frame deletion of *tssB* | This study |
| TssC-F  TssC-R | AGCGAAGGCAATACCCAGAC  GGTGCCGTACTCTTCTTCGT | 423 | *tssC* | This study |
| TssC-Q-F  TssC-Q-R | CTATGACATGATTACGAATTCCGTTGACCGCCACAAAATCC  GCCCGCAGGGCTTTAGGTCAGGCCATGGTTAATCCTCCGC | 987 | In-frame deletion of *tssC* | This study |
| TssC-H-F  TssC-H-R | TGACCTAAAGCCCTGCGGG  ACGACGGCCAGTGCCAAGCTTGTGCAGGATAAATGCCACGG | 998 | In-frame deletion of *tssC* | This study |
| TssD-F  TssD-R | CGACGAGATCAAAGG  TTCAGCGACAGGGTTTCGAC | 366 | *tssD* | This study |
| TssD-Q-F  TssD-Q-R | CTATGACATGATTACGAATTCAGCAAATACCTGTGGGGCAA  GGCCGTCCTTAACTGGCTTTCATCGCGTGTAGCTCCTGCA | 864 | In-frame deletion of *tssD* | This study |
| TssD-H-F  TssD-H-R | AAAGCCAGTTAAGGACGGCC  ACGACGGCCAGTGCCAAGCTTCAGGTGCGAAAGTGGATGGT | 656 | In-frame deletion of *tssD* | This study |
| TssE-F  TssE-R | GTTACCCCCGACCCTGCTG  GAATCGCTCATGGCTCACCT | 283 | *tssE* | This study |
| TssE-Q-F  TssE-Q-R | CTATGACATGATTACGAATTCTAGCAGCCTGACCTAAAGCCCT  AGTCGAGCAGTTGCGGGTCACCCTGTGCCGGACATGACG | 656 | In-frame deletion of *tssE* | This study |
| TssE-H-F  TssE-H-R | TGACCCGCAACTGCTCGACT  ACGACGGCCAGTGCCAAGCTTGCGCCACTTAGCACTTTGAT | 663 | In-frame deletion of *tssE* | This study |
| TssF-F  TssF-R | TGGTCAACGCCGTGCTTTATG  GCGTGCAGCCCAGAGCAATAT | 333 | *tssF* | This study |
| TssF-Q-F  TssF-Q-R | ACGACGGCCAGTGCCAAGCTTATCACCTACACGCTGGATAACG  CATGGCTCACCTCCATGCCT | 667 | In-frame deletion of *tssF* | This study |
| TssF-H-F  TssF-H-R | AGGCATGGAGGTGAGCCATGCTTTAAGCGCGACCCTGC  CTATTGACATGATTACGAATTCCACCAGGAAGTGTTGGCGGA | 619 | In-frame deletion of *tssF* | This study |
| TssG-F  TssG-R | CGCCTCCAGCCAGATTGAA  CACGGGCACCAGGAAGTGTT | 427 | *tssG* | This study |
| TssG-Q-F  TssG-Q-R | ACGACGGCCAGTGCCAAGCTTACCTGGATGATGCCGAAGAGATCCAGTAGAGCAAGCGTCATTGAGGCATACGACTTCCCTACC | 724 | In-frame deletion of *tssG* | This study |
| TssG-H-F  TssG-H-R | ATTGTCTGATGGAGCTTGGCA  ACGACGGCCAGTGCCAAGCTTACGCCGCAGCAGAATGTCAAT | 672 | In-frame deletion of *tssG* | This study |
| TssH-F  TssH-R | ACTAATCCTCGACACCCCCA  CGTCCCAGTCACCTGTTTCA | 313 | *tssH* | This study |
| TssH-Q-F  TssH-Q-R | CTATGACATGATTACGAATTCTTCGCCTCCAGCCAGATT  GCCAAGCTCCATCAGACAAT | 866 | In-frame deletion of *tssH* | This study |
| TssH-H-F  TssH-H-R | ATTGTCTGATGGAGCTTGGCGGCTGAGTGGGTATTGGAGT  ACGACGGCCAGTGCCAAGCTTAATACGAGCAGAACTGGCAA | 400 | In-frame deletion of *tssH* | This study |
| TssJ-F  TssJ-R | TGTGGTCGCAGGCTGTTCG  GCAGGGTCATTTCACGGCTAT | 395 | tssJ | This study |
| TssJ-Q-F  TssJ-Q-R | ACGACGGCCAGTGCCAAGCTTTTCCTGGAGCAGCTTGTCCA  TTATCGAGTCAGTGCCGCTACGCGGTCATGATTGTCCAAA | 650 | In-frame deletion *of* *tssJ* | This study |
| TssJ-H-F  TssJ-H-R | TAGCGGCACTGACTCGATAA  CTATGACATGATTACGAATTCCACGTAGGGCAACTGGATAAA | 605 | In-frame deletion of *tssJ* | This study |
| TssJ-C-F  TssJ-C-R | TAAGAAGGAGATATACATATGACCGCGAAACAGTTCAA  CAGGTCGACTCTAGAGGATCCCTACTCAATCGCTTTTTCGTCGTG | 474 | the complement of ∆*tssJ* | This study |
| TssK-F  TssK-R | CGGCTGGTGATCGGTTTACG  TCAGGCGGACTCTTGGGTGTTA | 324 | *tssK* | This study |
| TssK-Q-F  TssK-Q-R | CTATGACATGATTACGAATTCATCGCATTCCCTTGCTTGAC  ACGACTTCCCTACCGTATCACACGCTCATAGTTATCCTTCACATC | 725 | In-frame deletion of *tssK* | This study |
| TssK-H-F  TssK-H-R | TGATACGGTAGGGAAGTCGTATGC  ACGACGGCCAGTGCCAAGCTTCGCCAGAACAGATGACCGAC | 739 | In-frame deletion of *tssK* | This study |
| TssL-F  TssL-R | GGTGGATGAGACCCTGCTGTT  TGCGGCTGCTCCGTAGTTG | 507 | *tssL* | This study |
| TssL-Q-F  TssL-Q-R | ACGACGGCCAGTGCCAAGCTTACCTGGATGATGCCGAAGAGAT  CCAGTAGAGCAAGCGTCATTGAGGCATACGACTTCCCTACC | 724 | In-frame deletion of *tssL* | This study |
| TssL-H-F  TssL-H-R | CAATGACGCTTGCTCTACTGG  CAATGACGCTTGCTCTACTGG | 663 | In-frame deletion of *tssL* | This study |
| TssM-F  TssM-R | TGCTGCTGGAGCCCGTTT  AGCCGTTTCGTCTCGTCATTG | 391 | *tssM* | This study |
| TssM-Q-F  TssM-Q-R | CTATGACATGATTACGAATTCCCCCTGGCAAGTGATTCTGT  TCATTGCGGCTGCTCCGTA | 837 | In-framedeletion of *tssM* | This study |
| TssM-H-F/R  TssM-H- R | TACGGAGCAGCCGCAATGACATGAACCAGCTATCCGCCC  ACGACGGCCAGTGCCAAGCTTGCACTGGGTCAGTTGGCATA | 851 | In-framedeletion of *tssM* | This study |
| TssM-C-F  TssM-C-R | TAAGAAGGAGATATACATATGACGCTTGCTCTACTGGGCTGG  CAGGTCGACTCTAGAGGATCCTCAAGCAGCGAACTGACCCG | 3849 | the complement of *∆tssM* | This study |
| Hcp-wt-F  Hcp-wt-R | TTTAAGAAGGAGATATACATATGGATGCGATCATTCTCGA  CAAGCTTGCATGCCTGCATCACTTACCCAGGCGGTTCATTTCGATATCAGTGTAACTGGCTTTCTTGTTGGCG | 531 | Western blot | This study |
| GFP-F  GFP-R | CGCCACAACATTGAAGA  TGTAATCCCAGCAGCAGT | 189 | GFPuv from Pdsk-GFPuv | This study |
| gyrB-RT-F  gyrB-RT-R | ACCCGAACGAAGCCAAAGC  ATCCGCCAGCAGAGTCCC | 201 | PsaqRT-PCR  Reference gene | This study |
| dusA-RT-F  dusA-RT-R | ATATCGCACCAAACCCCAAT  TGACGCAAGAAACGCTCACG | 190 | Psa qRT-PCR  Reference gene | This study |
| ftrA-RT-F  ftrA-RT-R | CTCGTCGTCGCCCTCATCTAT  GTTTTCCAGCAGCACCTCACC | 195 | Psa qRT-PCR  Reference gene | This study |
| hrpR-RT-F  hrpR-RT-R | CGCTTCGTACTCGGCTTCCC  CGCTGCAACTCCCAGTTCTTTC | 213 | *hrpR* | This study |
| hrpL-RT-F  hrpL-RT-R | TGCTCAGGGCGTTTATCCA  AGCCAGGTCTGCGGTTTACT | 130 | *hrpL* | This study |
| hrcC-RT-F  hrcC-RT-R | TCAGGCAAGGCCAATATCC  CAGCTCCTTGACCAGTTTCT | 223 | *hrcC* | This study |
| hrpZ-RT-F  hrpZ-RT-R | GATCAAGGATGCTGGTGGTAA  GGCCTGGTTAGTCTGGTTATT | 204 | *hrpZ* | This study |
| hopM1-RT-F  hopM1-RT-R | GGACGGGCTGAATGAAAGTA  GTCCATCTCAAGAACGAGGTTAT | 120 | *hopM1* | This study |
| hopH1-RT-F  hopH1-RT-R | GAGGAAGCACTTGACCATATCA  GCTCAGAACAGCTTCGGTATAA | 150 | *hopH1* | This study |
| hopP1-RT-F  hopP1-RT-R | GGCCCGACAAGGTCAATAAA  CCTGTCCGCTTTCGATGATT | 134 | *hopP1* | This study |
