# Supplementary material for: Role of the Type VI Secretion System in the Pathogenicity of Pseudomonas syringae pv. actinidiae, the Causative Agent of Kiwifruit Bacterial Canker
Source: Front Microbiol. 2021 Feb 19;12:627785. doi: 10.3389/fmicb.2021.627785 (PMC7933208; doi:10.3389/fmicb.2021.627785)
Supplement: Supplementary file 2 [file Data_Sheet_1.DOCX]

**Supplementary file 1:**

**
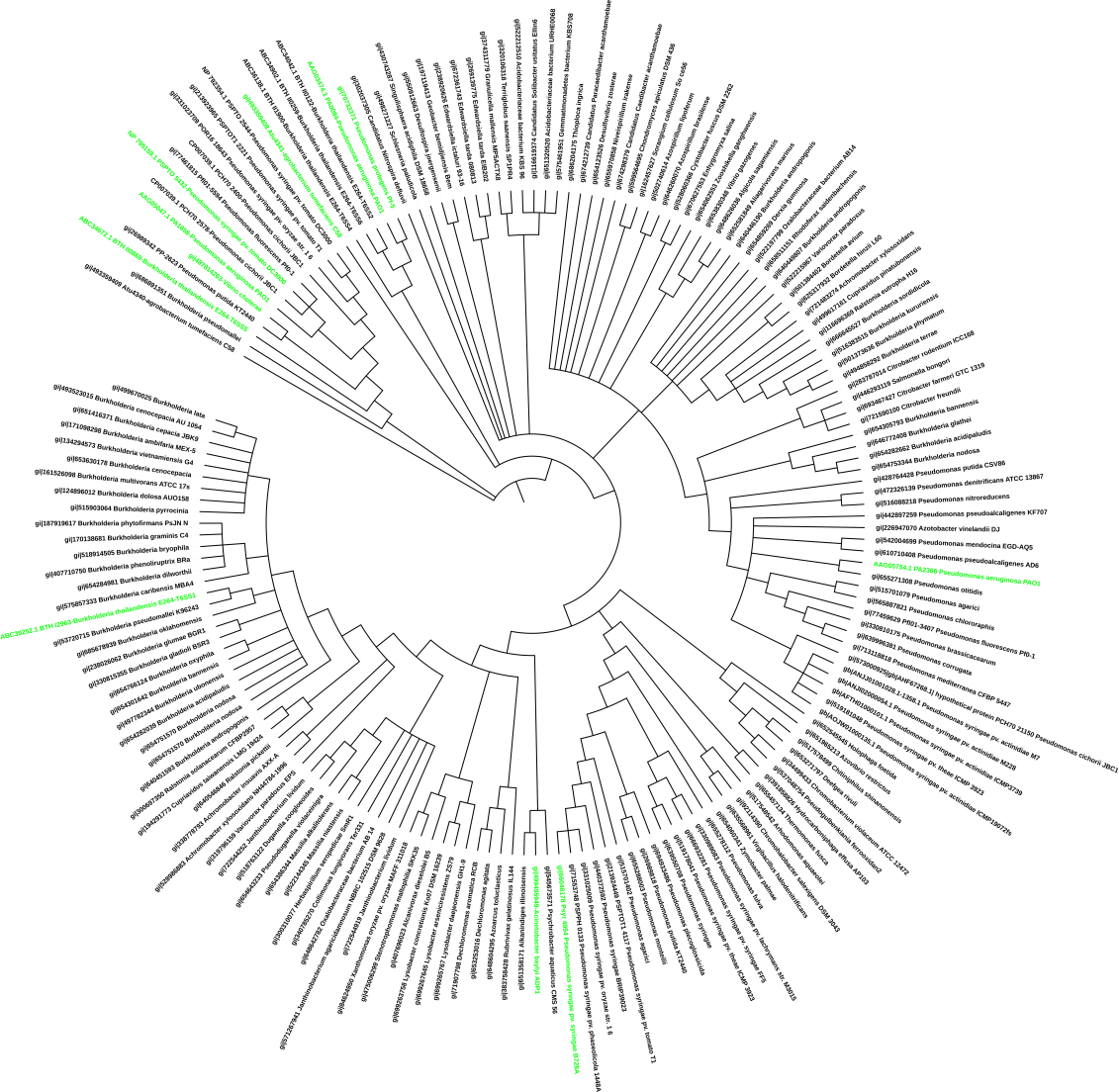
**

**Fig.S1 Evolutionary tree of pathogenic strain Psa M228 constructed by Maximum Likelihood method according to the *tssC* sequence of 68 species in 38 genera**

The whole genome of *Psa* M228 has been sequenced by our laboratory, and the genome data has been registered on NCBI (ANJI00000000.2). The gene *tssC* sequence of *Psa* M228 was obtained through bio-informatics analysis, and the evolutionary tree was constructed from the *tssC* sequence of 68 species in 38 genera, which have been reported to contain T6SS. According to the results, *Psa* M228 belonged to the same branch with the *P. aeruginosa* PAO1 H3-T6SS (HIS-Ⅲ) gene cluster in evolution. Evolutionary analyses were conducted in MEGA6.
